# Supplementary material for: An interdependent Cbf1–CCAN interaction stabilizes the budding yeast kinetochore
Source: Proc Natl Acad Sci U S A. 2026 Aug 4;123(32):e2610808123. doi: 10.1073/pnas.2610808123 (PMC13462599; doi:10.1073/pnas.2610808123)
Supplement: Supplementary file 1 [file pnas.2610808123.sapp.pdf]

PNAS

Supporting Information for
An interdependent Cbf1-CCAN interaction stabilizes the budding yeast kinetochore

Sabrine Hedouin, Changkun Hu, and Sue Biggins*

Sue Biggins
Email: sbiggins@fredhutch.org

This PDF file includes:

Figures S1 to S4
Tables S1 to S3
SI References

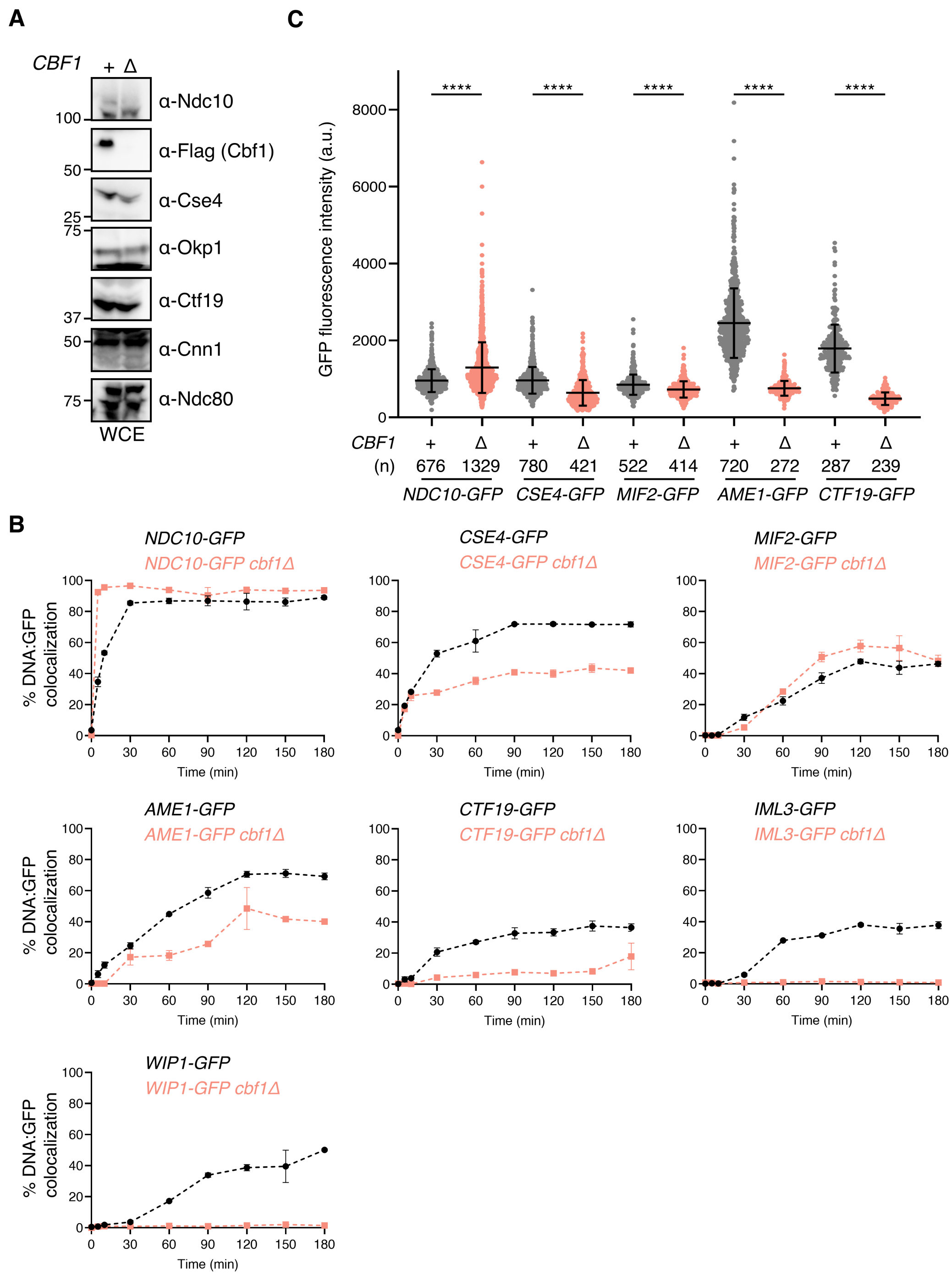

Fig. S1. Cbf1 is required for kinetochore assembly in vitro. (A) Whole cell extracts (WCE) from Fig. 1C were immunoblotted with the indicated antibodies. (B) Percentages of colocalization between *CEN3* DNAs and GFP-tagged protein in either in a wild type or *cbf1Δ* background as analyzed by TIRFM at various time points for 180 min. Strains used are as follows: *NDC10-GFP* (SBY22903), *NDC10-GFP cbf1Δ* (SBY24745*)*, *CSE4-GFP* (SBY22195), *CSE4-GFP cbf1Δ* (SBY24743), *MIF2-GFP* (SBY22094), *MIF2-GFP cbf1Δ* (SBY24615), *AME1-GFP* (SBY22119), *AME1-GFP cbf1Δ* (SBY24617), *CTF19-GFP* (SBY22116), *CTF19-GFP cbf1Δ* (SBY24619), *IML3-GFP* (SBY22199), *IML3-GFP cbf1Δ* (SBY24621), *WIP1-GFP* (SBY22207), and *WIP1-GFP cbf1Δ* (SBY24625). Error bars represent the standard deviation over three biological repeats. At least

3000 DNA molecules were imaged for each biological replicate. Data for GFP-tagged proteins in a wild-type background were replotted from (1). (C) Fluorescence intensity analysis of GFP-tagged proteins after 180 min of kinetochore assembly. The horizontal lines represent the mean, and error bars represent the standard deviation. The number (n) of total kinetochore foci analyzed is noted on the graph. Statistical significance was assessed using a two-tailed Mann–Whitney U test (****, $p<0.0001$).

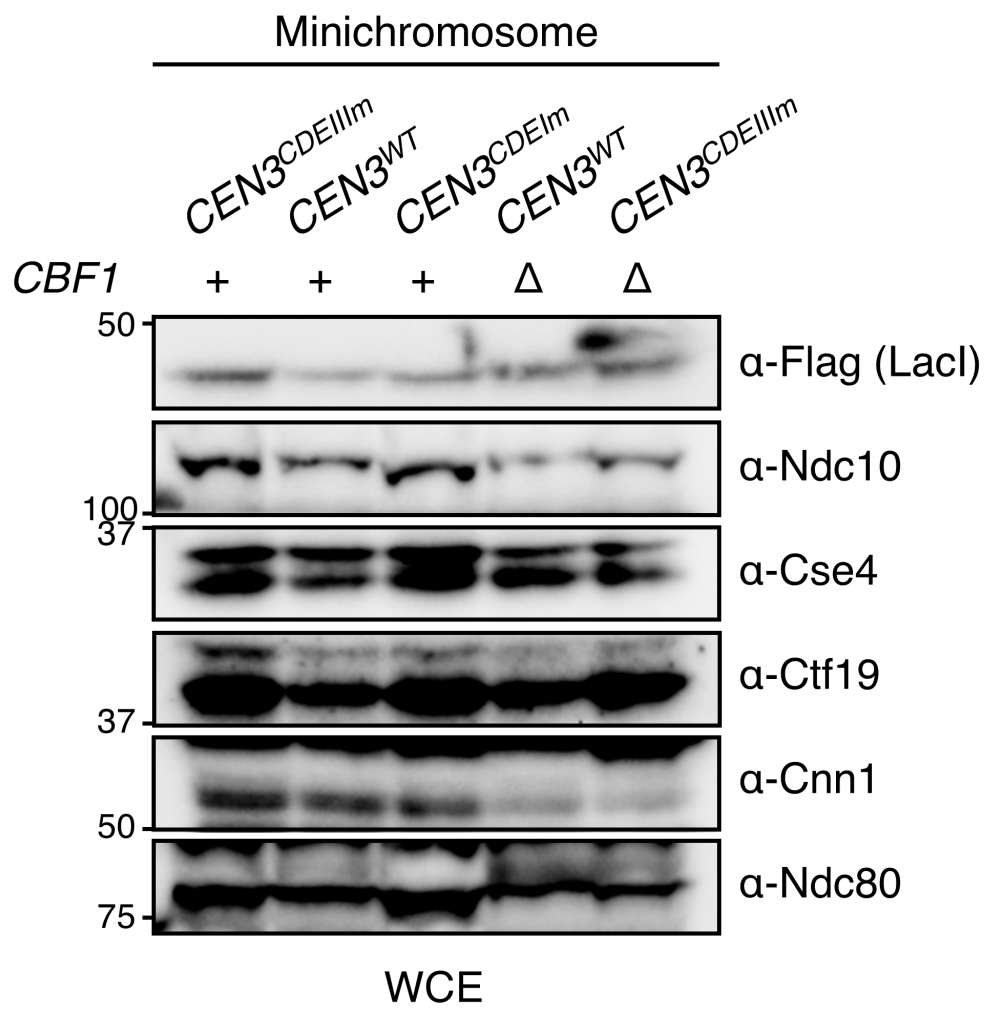

Fig. S2. Cbf1 is required for kinetochore assembly in vivo. Whole cell extracts (WCE) from Figure 2A were immunoblotted with the indicated antibodies.

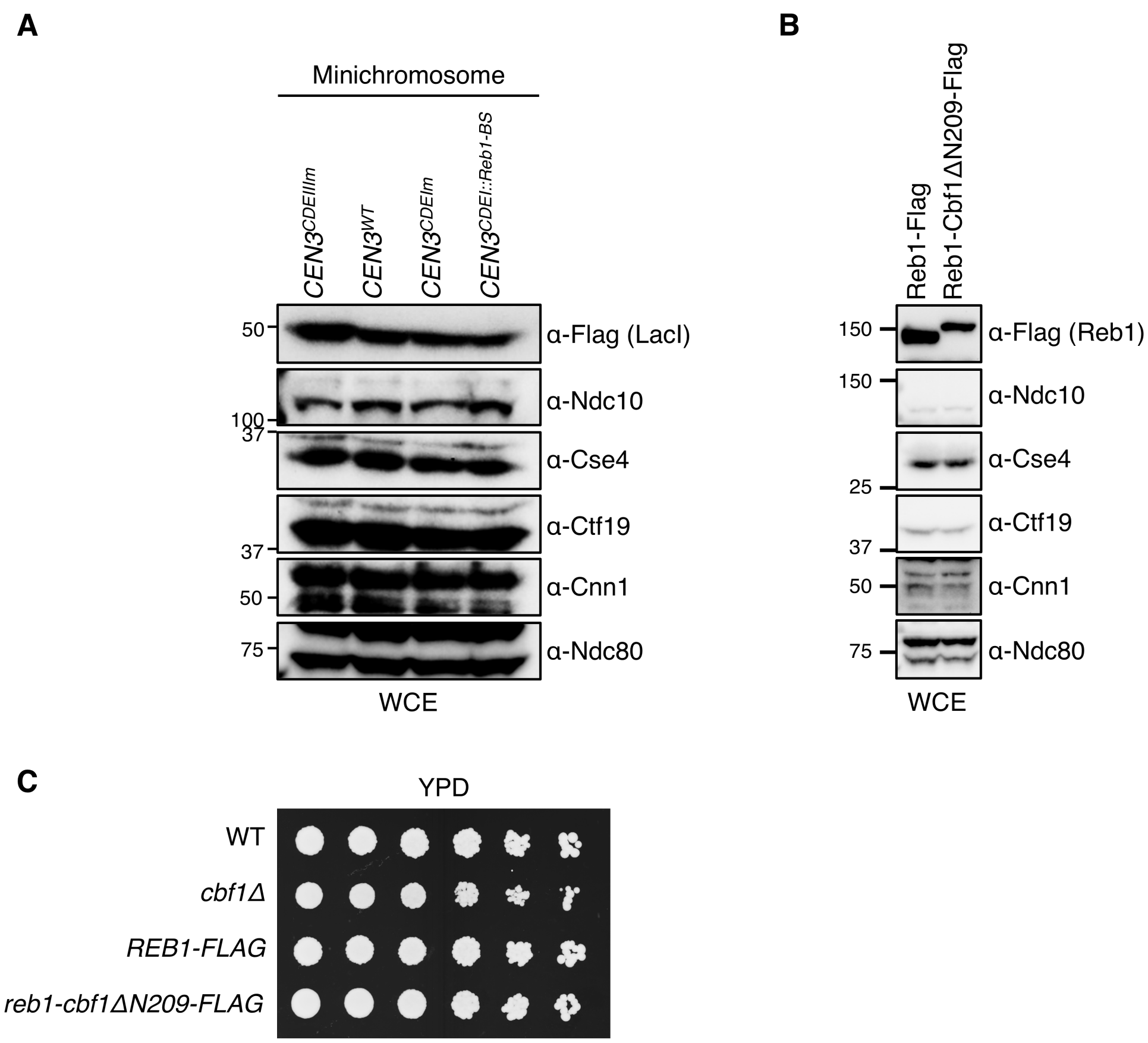

Fig. S3. Cbf1's role in CCAN recruitment is separate from its transcriptional roadblock activity. (A) Whole cell extracts (WCE) from Figure 3B were immunoblotted with the indicated antibodies. (B) Whole cell extracts (WCE) from Figure 3D were immunoblotted with the indicated antibodies. (C) 5-fold serial dilution series of WT (SBY3), *cbf1Δ* (SBY4958), *REB1-FLAG* (SBY25215), and *reb1-cbf1ΔN209-FLAG* (SBY25217) strains were spotted on YPD and incubated for 3 days at 23 °C.

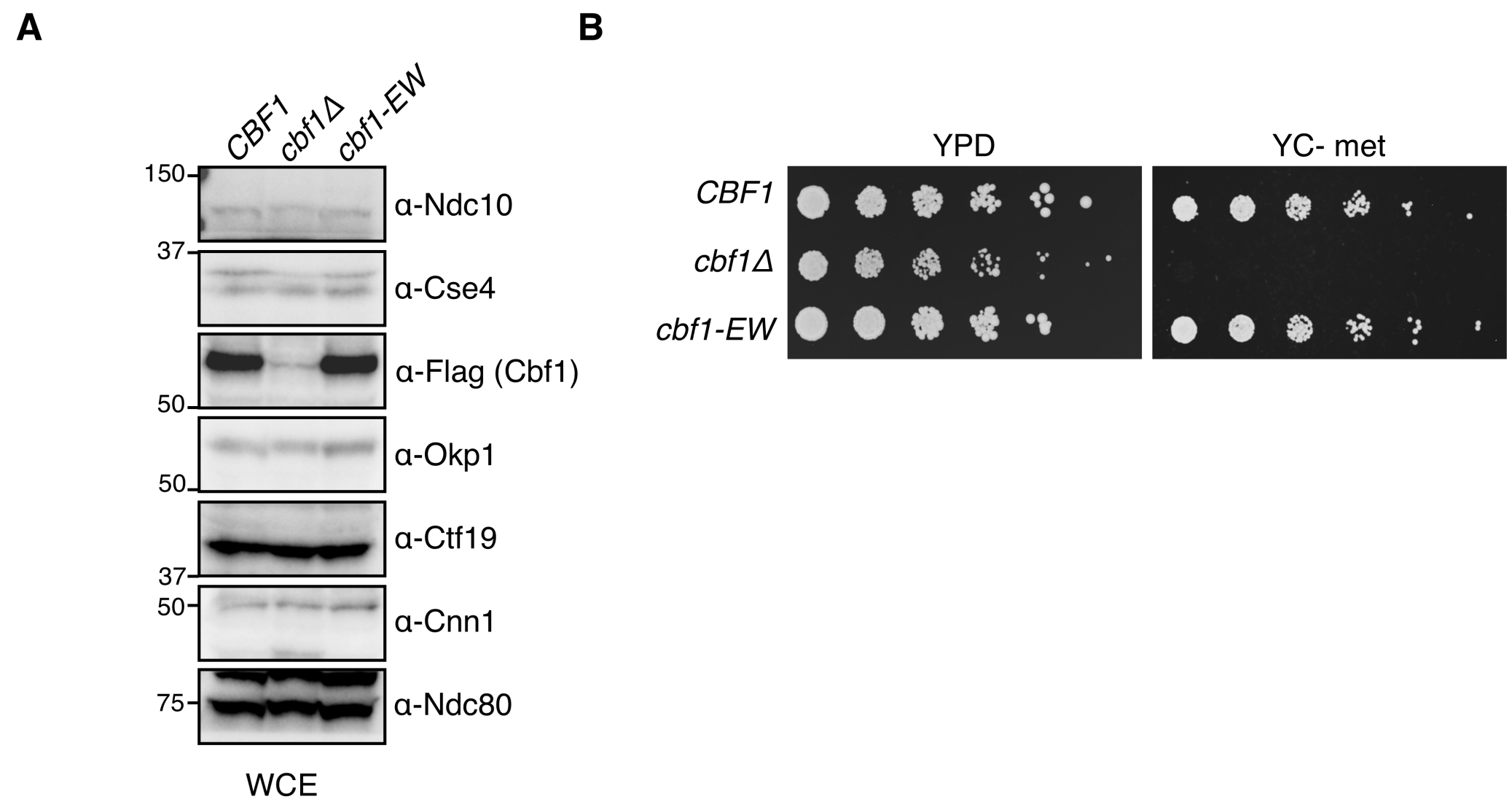

Fig. S4. Cbf1-EW mutant disrupts CCAN assembly and derepresses CEN transcription. (A) Whole cell extracts (WCE) from Figure 4C were immunoblotted with the indicated antibodies. (B) 5-fold serial dilution series of *CBF1-3FLAG* (SBY18421), *cbf1Δ* (SBY4958), and *cbf1-EW-3FLAG* (SBY22227) strains were spotted on YPD or YC-met (lacking methionine) plates and incubated for 3 days at 23 °C.

Table S1. List of *S. cerevisiae* strains used in this study

All strains are derivatives of SBY3 (W303)

Strain number	Genotype	Replicating plasmid
SBY3	*MATa ura3-1 leu2-3,112 his3-11 trp1-1 can1-100 ade2-1 bar1-1*	
SBY18421	*MATa CBF1-3xFLAG:TRP1*	
SBY4958	*MATa cbf1Δ::KanMX6*	
SBY22903	*MATα NDC10-GFP:KanMX6*	
SBY24745	*MATα NDC10-GFP:KanMX6 cbf1Δ::NAT*	
SBY22195	*MATα CSE4-GFP:NAT*	
SBY24743	*MATα CSE4-GFP:NAT cbf1Δ::KanMX6*	
SBY22094	*MATα MIF2-GFP:KanMX6*	
SBY24615	*MATα MIF2-GFP:KanMX6 cbf1Δ::NAT*	
SBY22119	*MATα AME1-GFP:KanMX6*	
SBY24617	*MATα AME1-GFP:KanMX6 cbf1Δ::NAT*	
SBY22116	*MATα CTF19-GFP:KanMX6*	
SBY24619	*MATα CTF19-GFP:KanMX6 cbf1Δ::NAT*	
SBY22199	*MATα IML3-GFP:KanMX6*	
SBY24621	*MATα IML3-GFP:KanMX6 cbf1Δ::NAT*	
SBY22207	*MATα WIP1-GFP:KanMX6*	
SBY24625	*MATα WIP1-GFP:KanMX6 cbf1Δ::NAT*	
SBY19145	*MATa ura3-1:pCMV-LACI-3FLAG:URA3 CNN1-3V5:KanMX CEN3-8LacO-TRP*	pSB963
SBY19146	*MATa ura3-1:pCMV-LACI-3FLAG:URA3 CNN1-3V5:KanMX CEN3*^{CDEIIIm}*-8LacO-TRP*	pSB972
SBY19147	*MATa ura3-1:pCMV-LACI-3FLAG:URA3 CNN1-3V5:KanMX CEN3*^{CDEIm}*-8LacO-TRP*	pSB2959
SBY19148	*MATa ura3-1:pCMV-LACI-3FLAG:URA3 CNN1-3V5:KanMX CEN3*$^{CDEI::Reb1-BS}$*-8LacO-TRP*	pSB3118
SBY19149	*MATa ura3-1:pCMV-LACI-3FLAG:URA3 CNN1-3V5:KanMX CEN3-8LacO-TRP cbf1Δ::KanMX6*	pSB963
SBY19150	*MATa ura3-1:pCMV-LACI-3FLAG:URA3 CNN1-3V5:KanMX CEN3*^{CDEIIIm}*-8LacO-TRP cbf1Δ::KanMX6*	pSB972
SBY24879	*MATa IML3-3mNeonGreen:HIS SPC110-mCherry::HPHMx*	
SBY24881	*MATa IML3-3mNeonGreen:HIS SPC110-mCherry::HPHMx cbf1Δ::KanMX6*	
SBY14171	*MATα dsn1-3A-3FLAG:URA3*	
SBY22227	*MATa cbf1-EW-3FLAG:TRP*	
SBY22129	*MATα CBF1-GFP:KanMX6*	
SBY22923	*MATα cbf1-EW-GFP:KanMX6*	
SBY24889	*MATα CBF1-GFP:KanMX6 ctf19Δ::KanMX6*	
SBY22452	*MATa CBF1-3xFLAG:TRP1 OKP1-3HA:HIS3*	
SBY22454	*MATa cbf1Δ::KanMX6 OKP1-3HA:HIS3*	
SBY22456	*MATa cbf1-EW-3xFLAG:TRP1 OKP1-3HA:HIS3*	
SBY25315	*MATa REB1-3FLAG:TRP1*	
SBY25317	*MATa REB1-cbf1ΔN209-3FLAG:TRP1*	

Table S2. List of plasmids used in this study

**Plasmid name**	**Relevant genotype**	**Marker**	**Use**	**Origin**
pSB963	*CEN3* (WT)	Ampicillin, TRP	Kinetochore assembly assay (*CEN3* WT and *ampC* templates)	(2)
pSB972	*cen3* (CDEIIIm)	Ampicillin, TRP	Kinetochore assembly assay (*CDEIIIm* template)	(2)
pSB2959	*cen3* (CDEIm)	Ampicillin, TRP	Kinetochore assembly assay (*CDEIm* template)	(3)
pSB3118	*cen3* (CDEI::Reb1-BS)	Ampicillin, TRP	Kinetochore assembly assay (*CDEI::Reb1-BS* template)	(3)
pSB3006	*CBF1-3FLAG:TRP*	Ampicillin	Template for fusing *cbf1-ΔN209-3FLAG:TRP* to *REB1* at its C-terminus	This study
pSB3744	*cbf1-EW-3FLAG:TRP*	Ampicillin	For replacement of endogenous *CBF1* locus with *cbf1-EW-3FLAG:TRP*	This study

Table S3. List of primers used in this study

Target	Primer name	DNA sequence (5' to 3')	Application	Comment / Purpose
CEN3	SB3878	BIOTIN-GGTTCTGGTGGTTCTGGTGGTTCTGGTGAATTCAAACAACCGCCGGCTTCCACCA	PCR	Amplification of CEN3 DNA from plasmids pSB963, pSB972, pSB2959, or pSB3118 to be used as template for the kinetochore assembly assay
	SB3880	ATCAGCGCCAAACAATATGGAA		
AME1	SB9009	GAAAAAAATAAATAAAATTAATGAAAATCTTTCTAACGAATTACAACCAAGTCTAGGTGACGGTGCTGGTTTA	PCR	Deletion of *CBF1* locus using pFA6a plasmids
	SB9010	AATACATATATACATATATATATATATATATATATACATCTTTTGAACCAATTCCTCGATGAATTCGAGCTCG		
CBF1	SB1058	CAACATCAAGTGCTTAAAATATAATACGGTTTTCTACACTTTTATTAACGCGGATCCCCGGGTTAATTAA	PCR	To tag *AME1* in C-ter using pFA6a plasmids
	SB1059	GCAGATACATAGGGAGACTCGAAATACATTTAGCTATCTATTTTTAACTCTCGATGAATTCGAGCTCGTT		
CBF1	SB5550	CGAAAGAAAAAGCACTAGGAGCGATAATCCACATGAGGCTAGGGAACAAAAGCTGGAGCT	PCR	To tag *CBF1* in C-ter using pFA6a plasmids
	SB5551	AGGGAGACTCGAAATACATTTAGCTATCTATTTTTAACTCCTATAGGGCGAATTGGGT		
CNN1	SB5152	TCCCTTAGAACTTCAATCAAGAATTGAAAGTTATTTGTTCCGGATCCCCGGGTTAATTAA	PCR	To tag *CNN1* in C-ter using pFA6a plasmids
	SB5153	AAATTGCCACTATTTAATTATTTTCTCTACGGTATCTTTTGAATTCGAGCTCGTTTAAAC		
CTF19	SB9027	AACCGGGTTAAAGGAGATCTGCAACGTTTGCCTATTCCCGGACATGTACGCCAGGGGTGACGGTGCTGGTTTA	PCR	To tag *CTF19* in C-ter using pFA6a plasmids
	SB9028	GAGCTTATCGGAATCGTTTAAGCAAGCCGTCCAGTTGGCAATGGCAAATGGAACATCGATGAATTCGAGCTCG		
IML3	SB9043	TCTCAGGAACAGAGTTCTAGCAGTTGTACTCCAATCGATTCAGTTTACCAGCGAGGGTGACGGTGCTGGTTTA	PCR	To tag *IML3* in C-ter using pFA6a plasmids
	SB9044	AAAAAAGGTAGAGCTGTGGTTTTTTATTGTATCTTGGTGAATATTCTTTATAGTGTCGATGAATTCGAGCTCG		
MIF2	SB9015	AGACGCTAACGATGACAACGACAAAGAATTAGACAGTACGTTTGACACTTTTGGGGGTGACGGTGCTGGTTTA	PCR	To tag *MIF2* in C-ter using pFA6a plasmids
	SB9016	CCTAGTTATATTTCTTCAGTACATAGCATGCATAATGAGAATATTCACATCATAATCGATGAATTCGAGCTCG		
NDC10	SB9041	GTGGAGGCATGACCATCAAAATTCATTTGATGGTCTGTTAGTATATCTATCTAACGGTGACGGTGCTGGTTTA	PCR	To tag *NDC10* in C-ter using pFA6a plasmids
	SB9042	TATAAACATACATGTCGGTATCCCTATACGAAACAGTTTAAACTTCGAAGCTCCCTCGATGAATTCGAGCTCG		

REB1	SB6222	TGATTATTTTAGCTCCAATATTTCAATGAAAACAGAAAATCGGATCCCCGGGTTAATTAA	PCR	To tag *REB1* in C-ter using pFA6a plasmids
	SB6223	TTATTGAGTTTTTCGCTTTCACCAATTATATTTTCCGGAAGAATTCGAGCTCGTTTAAAC		
REB1	SB6266	TGATTATTTTAGCTCCAATATTTCAATGAAAACAGAAAATCCTACTACTTTGGCCACAAC	PCR	To tag *REB1* in C-ter with *cbf1-ΔN209-3FLAG:TRP*
	SB6267	TTATTGAGTTTTTCGCTTTCACCAATTATATTTTCCGGAAGGCAAGTGCACAAACAATAC		
WIP1	SB9039	GGAAGTATATCCTTATCATATTGAAGCTGCAACGCAGGCTTTTCTGGATAGTCAAGGTGACGGTGCTGGTTTA	PCR	To tag *WIP1* in C-ter using pFA6a plasmids
	SB9040	TTATTTGCTATTACGAACAAAAGAGTATATGATAAAGAGGCTTAAAAATACCCCTTCGATGAATTCGAGCTCG		
CBF1	SB5798	ATTTAAATTTATGCTTTAGTATCGTCATATTC	Cloning	To clone *CBF1-3FLAG:TRP* into a pCRII-TOPO vector
	SB5799	ATTTAAATAAAGACATATTTGAAAGTCCGTC		
CBF1	SB7917	ATTGTGGAGCGAGCAAAACGCATCG	Cloning	Site-directed mutagenesis of L283E and L287W on plasmid pSB3006
	SB7918	TTTTGTTCCGTCCACTTTTCGATGTTTGC		
CEN8	SB7232	AACTCCAACAATTACACATCCACAAAACG	RT-qPCR	To amplify cenRNA from *CEN8*
	SB7233	TTTCTAAGTTCGGAACACAAAACCCAATG		
CEN4	SB6685	GATTACCGAAACATAAAACCTGCTCAAG	RT-qPCR	To amplify cenRNA from *CEN4*
	SB6795	TATGAAAGCCTCGGCATTTTGGC		
CEN5	SB6721	AGCAGTATTAGATTTCCGAAAAGAAAAAAAGG	RT-qPCR	To amplify cenRNA from *CEN5*
	SB6457	ACAACTGCTATTTATGTGCGGC		
UBC6	SB4137	GATACTTGGAATCCTGGCTGGTCTGTCTC	RT-qPCR	To amplify UBC6 transcript to be used as a RT-qPCR normalizer
	SB4138	AAAGGGTCTTCTGTTTCATCACCTGTATTTGC		

SI References

1. C. Hu, A. R. Popchock, A. A. Latino, C. L. Asbury, S. Biggins, Direct observation of interdependent and hierarchical kinetochore assembly on individual centromeres. *Nucleic Acids Res* **53**, gkaf1038 (2025).

2. B. Akiyoshi, C. R. Nelson, J. A. Ranish, S. Biggins, Quantitative proteomic analysis of purified yeast kinetochores identifies a PP1 regulatory subunit. *Genes Dev.* **23**, 2887–2899 (2009).

3. S. Hedouin, G. A. Logsdon, J. G. Underwood, S. Biggins, A transcriptional roadblock protects yeast centromeres. *Nucleic Acids Res* **50**, 7801–7815 (2022).
